# Value added by *Spirulina platensis* in two different diets on growth performance, gut microbiota, and meat quality of Japanese quails

**DOI:** 10.14202/vetworld.2016.1287-1293

**Published:** 2016-11-23

**Authors:** Mohamed S. Yusuf, Marwa A. Hassan, Mohamed M. Abdel-Daim, Adel S. El Nabtiti, Ali Meawad Ahmed, Sherief A. Moawed, Ahmed Kamel El-Sayed, Hengmi Cui

**Affiliations:** 1 Institute of Epigenetics and Epigenomics, Yangzhou University, Yangzhou, 225009 Jiangsu, China; 2 Department of Nutrition and Clinical Nutrition, Faculty of Veterinary Medicine, Suez Canal University, 41522 Ismailia, Egypt; 3 Department of Animal Hygiene, Zoonoses and Behavior, Faculty of Veterinary Medicine, Suez Canal University, 41522 Ismailia, Egypt; 4 Department of Pharmacology, Faculty of Veterinary Medicine, Suez Canal University, 41522 Ismailia, Egypt; 5 Department of Animal Wealth Development, Faculty of Veterinary Medicine, Suez Canal University, 41522 Ismailia, Egypt; 6 Department of Food Hygiene, Faculty of Veterinary Medicine, Suez Canal University, 41522 Ismailia, Egypt; 7 Department of Animal Wealth Development, (Biostatistics) Faculty of Veterinary Medicine, Suez Canal University, 41522 Ismailia, Egypt; 8 Department of Anatomy and Embryology, Faculty of Veterinary Medicine, Suez Canal University, 41522 Ismailia, Egypt

**Keywords:** isocaloric, isonitrogenous, meat quality, performances, quails, spirulina

## Abstract

**Aim:**

The growth promoting effect of the blue-green filamentous alga *Spirulina platensis* (SP) was observed on meat type Japanese quail with antibiotic growth promoter alternative and immune enhancing power.

**Materials and Methods:**

This study was conducted on 180 Japanese quail chicks for 4 weeks to find out the effect of diet type (vegetarian protein diet [VPD] and fish meal protein diet [FMPD])- Spirulina dose interaction (1 or 2 g/kg diet) on growth performance, gut microbiota, and sensory meat quality of growing Japanese quails (1-5 weeks old).

**Results:**

Data revealed improvement (p<0.05) of weight gain, feed conversion ratio and European efficiency index due to 1, 2 g (SP)/kg VPD, and 2 g (SP)/kg FMPD, respectively. There was a significant decrease of ileum mean pH value by 1 g (SP)/kg VPD. Concerning gut microbiota, there was a trend toward an increase in *Lactobacilli* count in both 1; 2 g (SP)/kg VPD and 2 g (SP)/kg FMPD. It was concluded that 1 or 2 g (SP)/kg vegetarian diet may enhance parameters of performance without obvious effect on both meat quality and gut microbiota. Moreover, 1 and/or 2 g (SP) may not be invited to share fish meal based diet for growing Japanese quails.

**Conclusion:**

Using of SP will support the profitable production of Japanese quails fed vegetable protein diet.

## Introduction

High performance, low mortality and thus profitable productions are continuing challenges facing poultry producers. Many ways are held to achieve that goal using growth promoting organic feed additives, dietary manipulation, and/or both strategies [1-4]. The bio additives can furnish to the nutrient requirements, activate the endocrine system and intermediate nutrient metabolism that help in improving animal yield [5,6]. One of the most important natural feed supplements for human and many animal species is the alga. *Arthrospira (Spirulina) platensis* is blue-green microalgae representative filamentous, non-N2-fixing cyanobacterium that has a great potential to enhance the food supply and possesses several valuable physiological features [7]. Due to its powerful content of valuable and high protein, essential amino acids (AAs), multi-vitamins and minerals, vital fatty acids and polysaccharides, SP considered as patented non-antibiotic substitutes as animal feed supplement having growth promoting as well as antioxidant effects in many species, especially poultry [6-9]. The application of microalgae as a supplement has been advised to profit on domesticated fowl including growth performance, livability, feed consumption, and carcass yield and quality. Recently, many authors concluded that SP supplementation improved body weight gain (BWG), feed conversion ratio (FCR), and increases the villi height [10].

Egg and meat type quails have gained much more popularity among consumers and sharing chicken meat in the Egyptian markets [11]. For rapid growth and good-quality meat of growing Japanese quails, dietary protein must not <24% [12] with balanced essential AA. Slips in the dietary protein may weaken both economic and productive poultry performance [13]. That high percentage may be achieved with the fish meals; however, high-cost pushes producers to formulate only soybean-corn based diets with some additives like alga to optimize essential AA [14,15].

Recent strategies essentially had been developed to use natural feed additives such as beneficial microorganisms or substrates with non-digestible ingredients, which are known as probiotic and prebiotic as alternative to use of antibiotics as growth promoters which had been banned in 1999 by the European Union (European Commission) [16] because of their destructive impacts, including microbial resistance to antibiotics; residues in chicken meat which might be harmful to human health and expansion of pathogenic microorganisms [17,18]. Spirulina can be considered as feed supplement that has many health benefits for humans, and a feed additive for animals having economic benefits [17]. SP is one of the most important micro-alga showing antimicrobial activity against many pathogenic bacteria and fungi [19] and it contains active ingredients such as tocopherols, C-phycocyanin, and extracellular polysaccharide [20-22] which have antimicrobial activities against *Escherichia coli*, *Pseudomonas* sp., *Enterobacter* sp., *Salmonella* *Typhi*; *Klebsiella pneumoniae*, and *Proteus vulgaris* [23-26]. *Staphylococcus aureus*; *Staphylococcus epidermis* and Aeromonas liquefaciens [27, 28]. Knowledge of these SP levels versus dietary types acts as economic alarm for producers who using fish meal based diet, helping them to choose the profitable way, especially when comparing among organic feed additives. Hence, our research question is: Which is an optimum dose of SP (1 g and 2 g) with VPD or FMPD will enhance growth performance parameters, gut microbiota and meat quality of growing Japanese quails from 1^st^ week old until 5^th^ weeks old.

## Materials and Methods

### Ethical approval

The study was conducted according to ethical guidelines approved by ethics of scientific research committee, Faculty of Veterinary Medicine, Suez Canal University, Ismailia, Egypt.

### Experimental birds, housing, and management

180 physically healthy, week old Japanese quail chicks were obtained in June 2013 from Agricultural Technological Center, Cairo University, Giza, Egypt. Birds of a week old were divided into six groups, 3 replicates with 10 birds, each housed in wired battery cages. Feeders and drinkers were supplied and cleaned daily with optimum temperature and ventilation, which were maintained using exhaust fans. Chicks were allowed *ad libitum* access to feed and water [29].

### Spirulina powder and chemicals

Pure premium SP powder was purchased from HerbaForce, UK (Table-1). Other chemicals used were analytical grade.

**Table 1:** Analytical chemical and nutritional composition of *S. platensis*^a^.

Constituent	Amount	Unit
Moisture	4-9	% DM
Crude protein	60-70	% DM
Fat (Mojonnier extraction)	4-16	% DM
Crude fiber	3-7	% DM
Mineral matter	3-11	% DM
Carbohydrate (total)	14-19	% DM
Minerals calcium	1200	mg/kg
Total phosphorus	13,000	mg/kg
Magnesium	3300	mg/kg
Potassium	26,000	mg/kg
Sodium	22,000	mg/kg
Fatty acids palmitic (16:0)	25.8-44.9	% of total fatty acids
Palitoleic (16:1 omega-6)	2.3-3.8	% of total fatty acids
Stearic (18:0)	1.7-2.2	% of total fatty acids
Oleic (18:1 omega-6)	10.1-16.6	% of total fatty acids
Linoleic (18:2 omega-6)	11.1-12.0	% of total fatty acids
Gamma-linoleic (18:3 omega-6)	17.1-40.1	% of total fatty acids
Vitamins/carotenoids-carotene	140,000	g/100 g
Total carotenoids	1700	mg/kg
Provitamin A	2,330,000	IU/kg
Thiamine (B1)	34-50	mg/kg
B2	30-46	mg/kg
Niacin (B3)	130-150	mg/kg
B6	5-8	mg/kg
B12	1.5-2.0	mg/kg
Foliate	0.50	mg/kg
AAs aspartate	5.20-6.00	% DM
Glutamate	7.04-7.30	% DM
Tyrosine	2.60-3.42	% DM
Methionine	1.30-2.75	% DM
Leucine	5.90-8.37	% DM
Phenylalanine	2.60-4.10	% DM
Lysine	2.60-4.63	% DM

DM=Dry matter.

a Adopted from [8,9,30-32].

*S. platensi*=*Spirulina platensis*

### Basal diets and experimental design

The first group control A (Cont. A) was non-treated corn-soybean meal (SBM) based diet with the fish meal protein diet (FMPD), second one control B (Cont. B) corn-SBM based without fish meal was concedered the vegetarian protein diet (VPD) with no additives. The third and fourth ones were FMPD given 1 and 2 g SP, respectively. The fifth and sixth groups were VPD plus 1 and 2 g SP, separately. Body weights were measured weekly between 7:00 and 8:00 am using a digital balance (sensitivity 0.001). The two diets were isocaloric and isonitrogenous as shown in Table-2.

**Table 2:** Composition of isocaloric and isonitrogenous experimental diets.

Ingredient %	FMPD	VPD
Yellow corn (8.9%)*	56.20	56.81
Soybean meal (44.1%)*	31.85	33.20
Fish meal (60.5%)*	2	0.00
Corn gluten meal (62%)*	6	6.90
Wheat bran (15.7)*	1.35	0.00
Dicap	0.53	0.86
Lime stone	1.25	1.35
Lysine	0.14	0.19
Methionine	0.08	0.09
Salt	0.3	0.30
Premix^#^	0.3	0.30
Calculated composition		
CP%	24	24
ME	2900	2900
Calorie/protein	120.8	120.8

* Determined values of crude protein %.

# High mix premix, each 3 kg provide: Vitamin A - 12 mIU, vitamin D3-2 mIU, vitamin E - 1000 mg, vitamin K3-1000 mg, vitamin B1-1000 mg, vitamin B2-5000 mg, vitamin B6-1500 mg, vitamin - B12 10 mg, biotin - 50 mg, pantothenic acid - 10,00 mg, nicotinic acid - 30,000 mg, folic acid - 1000 mg, manganese - 60,000 mg, zinc - 50,000 mg, iron - 30,000 mg, copper - 4000 mg, iodine - 300 mg, selenium - 100 mg, cobalt - 100 mg, carrier (CaCO_3_) to 3 kg

### Performance parameters

Initial live weight, weekly and final live body weight (LBW), BWG, feed intake (FI), and FCR were calculated weekly and for the entire period (from 7 to 35 days of age).

### Performance index (PI) [33]

PI=LBW (kg)×100/FCR.

### European efficiency index (EEI) [34]

EEI=Livability × live weight in kg/length of fattening period in days × FCR ×100.

### Measurement of intestinal pH and meat sensory tests

At day 35, three birds/replicate were randomly taken fasted closely for 12 h and then individually weighed, slaughtered, feathered, and eviscerated. Duodenum, jejunum, and ileum contents were aseptically collected in 90 ml sterilized physiological saline then pH values were measured by digital pH meter [35].

### Dressed carcass weight

Slaughtered chicks were plucked, eviscerated for PM inspection, and carcasses were kept for 4 h at 4°C, then each carcass-without feet-was weighed (empty carcass weight). Carcass yield was calculated as the ratio of LBW [36]; after that 5 panelists from the department of meat and milk hygiene were invited to measure meat sensory parameters (odor and color).

### Enumeration of intestinal microflora

One gram of ileocecal contents of five slaughtered chicks from each group were collected aseptically at the end of the experiment, transferred into a sterile test tube containing 9 ml sterile nutrient broth and diluted by tenfold serial dilution (10^-1^ to 10^-5^) then 10 µl [37] of mixed diluted sample using vortex (WiseMix, VM-10, Korea) was plated using drop plate technique described by Herigstad *et al*. [38] for enumeration of total bacterial count (TBC), total coliform, total streptococci, total *Lactobacilli*, and total Staphylococci onto the following media: Standard plate count agar (Difco, USA), EMB agar (Lab M, LAB061, UK), Selective Streptococci agar (Biolife, Italia), De Man-Rogosa-Sharpe agar (MRS, Lab M, LAB093, UK), and Mannitol salt agar (Lab M, UK). All plates were cultivated at 37°C for 24-48 h while plates for *Lactobacilli* were incubated in 3% CO_2_ atmosphere at 37°C for 48-72 h. The results were expressed as arithmetical means±SE (in log cfu/g).

### Statistical analysis

Data of this study were analyzed using analysis of variance (ANOVA) procedures based on general linear models of factorial experiments. Two-way ANOVA procedures were used to account for the effect of both the main and interaction effects according to Snedecor and Cochran [39]. Means separation and pairwise comparisons were done by Duncan’s multiple range test [40]. Statistical analyses were conducted by SPSS for windows (SPSS version 20). Results are considered significant at probability level of (p≤0.05).

### Results and Discussion

First, PM inspection showed darker livers concerning 3^rd^ group SP1A with some hepatomegaly than 4^th^ group SP2A, no inflammatory signs were detected in the internal organ and inspected muscles. Data were recorded from 7^th^ to 35^th^ day old. Whereas FCR and BW are favorable correlated, data concerning final LBW and body WG together with FCR (Table-3). There was improved growth rate and feed efficiency (WG:FI) by selection of quail for live weight at 4 weeks of age [36,41,42], but we started the feeding trial at the 7^th^ day of age, because chicks were hatched in another province, 2 h needed for transportation and purchased at 4^th^ day old, so they held for rest and adaptation. The quails that subjected to 1 g SP in vegetarian diet 5^th^ group (SP1B) showed significant (p<0.05) highest LBW and body WG in comparison among other groups followed by 6^th^ group SP2B; these data were in accordance [10,43]. Normally, FMPD excelled significantly (p<0.05) the VPD diet in parameters of final LBW and body WG; also no positive effect as well as no significant depression was felt due to SP1A and SP2A on LBW and body WG when compared with control non-treated groups, that results supported findings by Toyomizu *et al*. [44] and Ross and Dominy [45] who used fish meal in his experimental diet but with higher levels of SP. Data revealed strong correlation between improved growth and FCR (Table-3) where the highest and significant effect (p<0.05) was obtained by SP1B followed by SP2B, also FMPD was continued to overpass the VPD significantly (p<0.05) regarding FCR, BWG and final BW; the results that supported by many authors who studied the response of birds to fish meal contained diets versus VP diets [46-48], in contrast many authors found no significant differences in the body WG of broilers fed VP diets or diets containing animal protein (pig and poultry byproducts). Poorer protein digestibility as well as energy utilization may be the cause of any differences between VP diets and diets containing animal protein [49,50]. Moreover, unidentified factors especially in fish meal were implemented behind the improvement of growth performance parameters of birds fed that diets [47].

**Table 3:** Initial weight, final weight, body weight gain (g) and FCR of experimental groups.

Diet type	FMPD			VPD		
	
	Control	*Spirulina* 1 g/kg	*Spirulina* 2 g/kg	Control	*Spirulina* 1 g/kg	*Spirulina* 2 g/kg
Initial weight (g)	21.43±0.73	21.07±0.20	21.87±0.41	20.50±0.90	21.20±0.49	22.47±0.34
Final weight (g)	202.2±0.79^c^	201.9±3.06^c^	203.5±1.53^bc^	192.3±0.78^d^	211.7±1.05^a^	208.2±2.06^ab^
Weight gain (g)	180.8±1.4^b^	180.8±2.9^b^	180.8±0.6^b^	171.8±0.8^c^	190.5±0.9^a^	185.8±1.8^a^
Feed intake (g)	628.8±2.1	608.3±3.2	587.5±2.7	629.3±1.4	596.6±1.3	608.5±6.9
FCR	3.47±0.04^b^	3.36±0.05^bc^	3.25±0.08^cd^	3.66±0.02^a^	3.13±0.06^d^	3.27±0.05^cd^

a-c Values are means±standard error (SE). Feed conversion was adjusted for mortality. Means within the same row with different superscripts are significantly different (p<0.05). FCR=Feed conversion ratio, FMPD=Fish meal based diet, VD=Vegetarian diet

The analytical chemical and nutritional composition of SP as shown in Table-1 were taken in concern to explain the unexpected data of using *Spirulina* with FMPD diets for growing Japanese quails. A homeostatic adaptation resulted due to marginal supplementation of any essential AA to prevent a large accumulation of the AA in blood and tissues. Unbalance may happen as results of higher available levels of SP methionine (Met) and lysine (Lys), AA together with highly digestible AAs of fish meal resulted in decreased FI (Table-3) in SP1A group, and birds were responded by decrease FI as a results of excessive intake of essential and nonessential AA [51-53]. That high blood level helping in oxidation of excess AA for (a) source of energy (much more energy also required to digest the animal protein for the target production [54] more than plant protein); (b) nitrogen excretion from the body [13], especially from kidney that may increase reactive species (RS) production due to slow catabolism of Met [55]. In addition, FI and WG of chicks given 1.44% Lys in their diets from 1 to 21 day of age were passively respond [56].

Heptadecane is a volatile component of SP, and blocks the *de novo* synthesis of fatty acids and ameliorates several oxidative stress-related diseases [57,58]. The potency of heptadecane to suppress the proinflammatory gene expressions of COX-2 and iNOS (both NF-kB-related genes) and RS production in aged kidney tissue was revealed by Kim *et al*. [58], that is may be the worst case scenario that resulted in weak kidney functions together with inefficient liver enzymes in SP1A group, compared SP2A group that may have acceptable performance parameters behind spirulina-supplemented VP diets, due to a higher level of heptadecane. That explanation may need further studies especially in light of epigenetic changes in some liver and kidney enzymes concerning anabolism and catabolism. Table-4 showed a significant (p<0.05) decrease in ileal pH which may indicate an improved protein digestibility correlated with higher performance in VP group, also there were non-statistical differences among the control, SP1B and SP2B groups, that supported the value of spirulina with VP diets for growing Japanese quails.

**Table 4:** PI, EEI, dressed weight %, meat quality and intestinal pH of experimental groups.

Diet type	FMPD			VPD		
	
	Control	*Spirulina* 1 g/kg	*Spirulina* 2 g/kg	Control	*Spirulina* 1 g/kg	*Spirulina* 2 g/kg
Performance index	5.82±0.05^c^	6.00±0.18^bc^	6.27±0.14^b^	5.25±0.03^d^	6.76±0.11^a^	6.36±0.16^b^
European efficiency index	2.51±0.04	2.43±0.01	2.36±0.07	2.5±0.01	2.37±0.05	2.43±0.02
Dressed carcass weight %	66.0±0.95	66.2±0.17	67.5±1.08	66.3±0.65	68.5±0.92	71.7±1.30
Color	26.3±0.88^a^	16.3±0.88^c^	24.3±1.76^ab^	27.3±0.88^a^	22.3±1.45^b^	22.3±0.33^b^
Odor	27.3±1.45	21.7±0.33	25.0±1.73	27.0±0.58	23.7±1.45	26.0±2.31
Duodenal pH	6.17±0.03	6.21±0.01	6.27±0.10	6.19±0.01	6.19±0.05	6.26±0.001
Jejunum pH	6.14±0.04	6.17±0.04	6.25±0.06	6.24±0.08	6.12±0.01	6.24±0.06
Ileum pH	6.27±0.05^a^	6.36±0.02^a^	6.53±0.15^a^	6.54±0.09^a^	5.57±0.18^b^	6.56±0.10^a^

a-c Values in the same row that have different superscripts are significantly different (p>0.05). FMPD=Fish meal based diet, VD=Vegetarian diet, PI=Performance index, EEI=European efficiency index

### Meat quality

Unbalanced dietary protein considered the starting point for activation of enzymes incorporated in AA catabolism; as deficiency increases the catabolism of tissue fat and protein from the poultry carcass whereas extra protein increases the oxidation of AAs as source of energy and nitrogen excretion from the body, so the protein concentration in commercial quails production should be optimized to keep the economic and productive performance [13,59]. AAs in excess in bloodstream are toxic to animal body, so quick removal is a must, using the liver, catabolism and excretion of nitrogen ingested above the body needs [60]. Birds fed on animal protein diets accumulated more abdominal fat than birds fed VP diets. Our findings (Table-4) can be used to advantage in diet formulation; when lean meat is desired, birds may be fed VP diets, which will result in less fat deposition and more protein deposition in the carcass. This will not only reduce fat waste at processing plants but also gives a better quality product for the consumer [47,61].

### Enumeration of intestinal microflora

The health and performance of poultry influenced greatly by gastrointestinal normal flora [62]. The effects of dietary supplements on counts of ileal-cecal microbiota (log cfu/g ileocecal content) are illustrated in Table-5. Results of TBC revealed unobvious effect in birds received FMPD as comparing with their control. On the other hand, birds received vegetarian protein diet showed a significant decrease SP2B when compared to VPD control. Furthermore, coliform count in both protein diets showed the same pattern as well as in TBC. Another indicator of pathogenic bacteria is the Staphylococci count which showed nonsignificant change in both doses of spirulina in the two diet types when compare each dose with the control type. Regarding beneficial bacteria (*Streptococci* and *Lactobacilli* counts) showed 2 different pattern, whereas the first one showed an improvement as direct relationship between streptococci count, and high dose of SP2A, which could be illustrated in the form of a significant (p≤0.05) increase as compared to its control, another form of improvement in streptococci count was observed in the form of non-significant increase in both doses of VPD. On the other hand, *Lactobacilli* count showed a significant (p≤0.05) decrease in SP1A and non-significant change in the other groups. These results showed that the supplementation of SP to vegetarian protein diet in a dose of 2 g/kg could be positively influenced the ileocecal microbiota which observed in form of a significant reduction in both total bacterial and coliform counts in addition to, trend toward increase in Streptococci count. Streptococcus species considered as good probiotic that can maintain balance between the beneficial and harmful pathogenic bacteria in the gut and also directly correlated with health promotion [63]. These results partially in agreement with Shanmugapriya *et al*. [18] who found that 1% of dietary SP might have a negative effect on *E. coli* as a main component of coliform. There was a conflict between previous researchers in their findings, whereas one team [64] had found no effect of administration of SP but they found no substantial effect on the composition of the cecal microbiota (TBC, *E. coli* and campylobacter) of rabbits fed 5% SP, and many investigators in the other team [18,63,65] found positive effect of dietary SP to poultry on improving lactobacillus count in intestine, and their results were in contrast to our finding whereas no positive effect was observed on *Lactobacilli* count and this could be referred to the different doses (as they used higher doses), experimental animals and the method of extraction of the algae. Further studied is needed for examination the effect of SP on quails with different doses.

**Table 5:** Bacterial count in ileocecal content in different groups.

Diet type	FMPD			VPD		
		
Log (CFU/g)	Control	*Spirulina* 1 g/kg	*Spirulina* 2 g/kg	Control	*Spirulina* 1 g/kg	*Spirulina* 2 g/kg
TBC	8.64^b^±0.2	8.76^b^±0.34	8.85^b^±0.04	9.66^a^±0.12	9.27^ab^±0.15	8.72^b^±0.1
Coliform	8.43^b^±0.13	8.15^b^±0.49	8.4^b^±0.07	9.25^a^±0.16	8.79^ab^±0.19	8.25^b^±0.12
Streptococci	6.7^c^±0.22	7.1b^c^±0.21	7.85^a^±0.13	7.69^ab^±0.31	8.24^a^±0.18	8.01^a^±0.14
*Lactobacilli*	8.3^b^±0.006	7.76^c^±0.32	8.53^b^±0.01	9.28^a^±0.13	8.69^b^±0.12	8.36^b^±0.08
Staphylococci	6.04^b^±0.06	5.88^b^±0.13	6.11^b^±0.14	7.03^a^±0.22	6.72^a^±0.19	6.78^a^±0.2

a-c Mean within row having different superscript are statistically significant (p≤0.05). FMPD=Fish meal based diet, VPD=Vegetarian protein diet

## Conclusion

The commercial production of Japanese quails (*Coturnix Coturnix japonica*) has increased during recent years. Knowledge of these SP levels versus dietary types acts as economic alarm for producers who using FMPD, helping them to choose the profitable way, especially while comparing organic feed additives.

## Authors’ Contributions

MSY, MAH, and ASE were equally contributed to concept and design the experiment along with data analysis and writing. Ali Meawad Ahmed directed the carcass quality analysis and interpretation MMA brings the *Spirulina* additives and helped in sampling. SAM directed and supervised the statistical analysis. HC supervised editing the manuscript. All the authors read and approved the final manuscript.
